# Open Achilles Tendon Rerupture: A Case Report and Review of the Literature

**DOI:** 10.1155/2020/6694968

**Published:** 2020-12-02

**Authors:** Michael E. Doany, Megan C. Paulus

**Affiliations:** Stony Brook University, Department of Orthopaedic Surgery, 101 Nicolls Road, HST T-18, Room 89, Stony Brook, NY 22794-8181, USA

## Abstract

In this report, we describe a rare case of open rerupture of an Achilles tendon following primary surgical repair. The rerupture occurred 12 weeks postoperatively and was associated with a transverse open wound perpendicular to the original surgical incision. This complication was successfully managed utilizing the preexisting transverse wound and a minimally invasive repair technique, minimizing further risk to the soft tissues overlying the tendon. This rare complication has only been described a few times in the literature and is likely associated with adhesions between the tendon repair and the subcutaneous tissues.

## 1. Introduction

Achilles rupture is a common injury with an incidence around 31 per 100,000, [1] typically occurring in the middle-aged healthy active population. Excellent outcomes are reported with both operative and conservative treatment; however, rerupture remains an important issue for the treating surgeon. Meta-analysis and randomized controlled trials have reported rerupture rates range from 2 to 12% [1–3]. Historically, nonoperative management was associated with significantly higher rates of rerupture, but with the introduction of early functional rehabilitation protocols, reported rates of rerupture have been found to be equivalent between treatment groups [1–3]. Operative groups do consistently show a higher overall rate of complication, generally due to infection and wound problems [1, 2, 4]. More recently, percutaneous techniques have become popular, with equivalent rerupture and potential lower incidence of deep infection, with a higher occurrence of sural nerve injury [5–9]. Several augmentation techniques have also been described, but have not been proven to improve outcomes or decrease rerupture rates [10, 11].

Rerupture of a surgically repaired Achilles tendon is uncommon, and there has been little written in regards to its management other than to describe inferior outcomes after rerupture [12–14]. In this report ,we describe an interesting case of an open rerupture after primary Achilles repair and review the few cases in the literature regarding open Achilles tendon reruptures.

## 2. Case Report

A 38-year-old healthy male presented to the office three days after feeling a pop behind his left ankle after a noncontact injury during a recreational basketball game, complaining of pain in his Achilles. The patient had been ambulating with a short pneumatic walker and crutches that he had at home. On the physical exam, he was noted to have moderate swelling and ecchymosis, tenderness along the Achilles tendon, and a palpable gap in the midsubstance of the Achilles tendon. With the patient in the prone position with the knees bent, the resting tension of the Achilles was decreased on the injured side. He had a positive Thompson test, and bedside ultrasound revealed a full-thickness defect with approximately 1 cm gapping with forced plantar flexion. The patient had no preexisting symptoms of tendinopathy, was a nonsmoker, and had no risk factors for complication. A discussion was had with the patient regarding operative versus nonoperative management of his injury, and decision was made to proceed with operative repair of his Achilles tendon.

**Figure 1:**
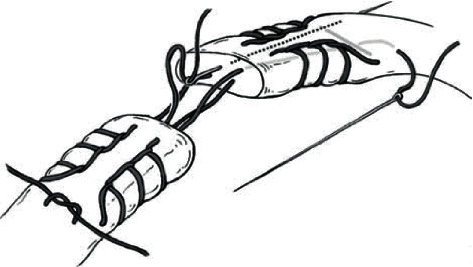
The gift box technique. Free suture ends passed across the rupture site to the opposite side of tendon and tied distal to the crossover stitch of the other suture (reproduced with permission from Sage Publishers, Adapted from Labib SA, Rolf R, Dacus R, Hutton WC. The “Giftbox” Repair of the Achilles Tendon: A Modification of the Krackow Technique. *Foot & Ankle International* 2009, 30(5)  : 410-414).

The patient was taken to the operating suite five days later for open Achilles tendon repair. He was positioned prone, and a midline incision was made over the Achilles tendon, dissecting sharply down through the paratenon. The Achilles rupture was identified, debrided, and repaired end to end with nonabsorbable suturetape® (Arthrex, Inc., Naples, FL) using a modified Krackow technique as described by Labib et al. [15]. Sutures were passed proximal and distal to the Krackow stitches, using the gift box technique and tied with the ankle held in maximal plantar flexion (Figure 1). The ruptured plantaris tendon was weaved through the Achilles tendon proximally and out distally for further reinforcement of the repair. A 2-0 braided absorbable suture was used to close the paratenon and the subcutaneous tissue. The skin was closed with a series of nylon sutures with a tensionless repair. The patient was placed in an AO plaster splint with the ankle positioned in plantar flexion. He was instructed to remain nonweight bearing for two weeks following surgery.

At 2 weeks postoperatively, the patient's incisions were healed, and his sutures were removed. He was transitioned out of his splint into a pneumatic walker with heel wedges at 2 weeks. He was instructed to begin gentle ankle range of motion exercises, in line with the functional rehabilitation protocol described by Willets et al. [3]. He was advanced to partial weight bearing in the CAM walker at 3 weeks and full weight bearing at 4 weeks. At 6 weeks postoperatively, the patient was sent to begin formal physical therapy, removed the heel wedges from the CAM walker, and transitioned to a sneaker by 8 weeks postoperatively.

By postoperative week 12, the patient's skin had completely healed, and he was ambulating without his pneumatic walker and was progressing well in physical therapy. He was walking up his stairs at home and felt a sudden pop in the back of his ankle at the surgical site. He had a wound in the prior surgical region and reported to an outside emergency department. He was found to have a transverse wound on his posterior ankle overlying the Achilles, perpendicular to his longitudinal surgical incision, with an associated rerupture of the Achilles tendon. The skin was repaired with suture at the emergency room, and the patient followed up in clinic several days later. At follow-up, it was noted that there was no continuity of the Achilles tendon with Thompson testing, and there was a palpable gap, along with a transverse laceration poorly reapproximated with serosanguinous drainage. The patient was indicated for irrigation and debridement with revision Achilles repair.

**Figure 2:**
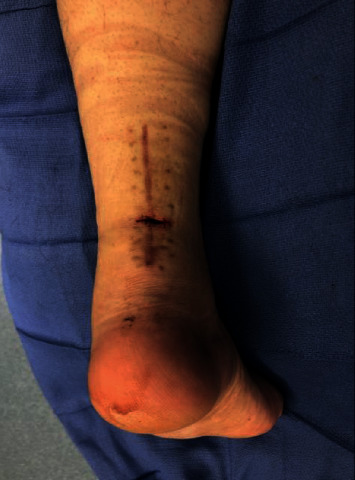
Intraoperative photograph of open rerupture of the achilles tendon. Axis of the wound is transverse and perpendicular to the original surgical incision.

**Figure 3:**
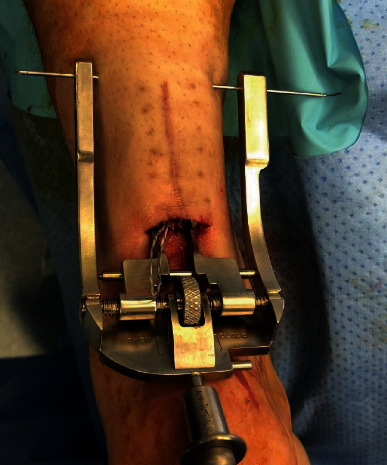
Revision surgical intervention utilizing minimally invasive jig to pass sutures through the proximal Achilles stump.

**Figure 4:**
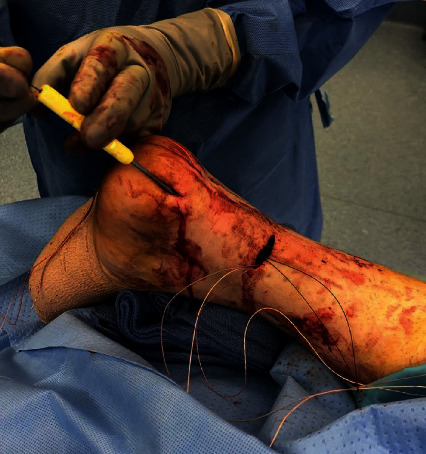
Sutures being passed from proximal Achilles through distal tendon stump with suture lasso to be anchored in the calcaneus.

The patient was taken back to the operating room and placed in prone position. A 2 cm transverse laceration over his prior Achilles rupture site was noted, with an underlying rerupture of the Achilles tendon at the same level (Figure 2). The incision was extended medially and laterally, and dissection was carried down to the tendon. The suture tape was identified, with knots still intact, but the sutures had pulled out of the tendon proximally. The suture tape was removed, and the area copiously irrigated; no evidence of infection was noted. A percutaneous jig (Arthrex, Inc., Naples, FL) was utilized in accordance with the PARS® midsubstance Achilles repair technique through the open transverse laceration (Figure 3). Sutures from the proximal Achilles stump were passed through the distal tendon with a lasso and secured into the calcaneus using swivel lock anchors® (Arthrex, Inc., Naples, FL) with the ankle held in plantar flexion (Figure 4). The same functional rehabilitation protocol was used postoperatively.

**Figure 5:**
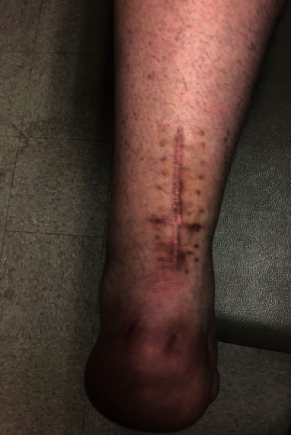
Six months postoperative visit from revision surgery.

At most recent follow-up, six months after revision surgery, the patient's skin is well healed, and he walks without a limp and has begun jogging again (Figure 5).

## 3. Discussion

Rerupture after Achilles repair is reported at about 2-12%, with no statistical difference reported between open, percutaneous, augmented, or closed treatment methods [1–4, 7, 9, 16]. Open rerupture is an exceedingly rare complication and has only been described a handful of times [17–19]. This report is the first case that we are aware of described in the literature of an open rerupture of the Achilles tendon successfully repaired utilizing a minimally invasive technique through the preexisting transverse laceration.

Garcia-German et al. were the first to describe open rerupture of an Achilles tendon associated with a transverse skin tear perpendicular to the surgical incision [18]. They reported on their experience with two cases of open rerupture and described a few technical factors likely related to this complication. The two cases described were repaired with a Bunnel type suture technique augmented by gastrocnemius-soleus turndown flap. In both cases, the bulk of their repair prevented complete closure of the paratenon. Additionally, a conservative postoperative protocol restricted the patients to nonweight bearing in a cast for 8 weeks. Both of these factors may lead to increased adhesions between the repaired tendon and subcutaneous tissue. The first rerupture occurred in postop week 12, when the patient began sprinting during rehab, being noncompliant with protocol. The second rerupture occurred in postop week 9, just two days after cast removal, when the patient stumbled on a step at home. Both patients went on to have open repair through a traditional longitudinal incision and were satisfied with their final results.

Hanada et al. described another two cases of open rerupture, both of which occurred traumatically early in the postoperative course [19]. In both of these cases, the paratenon was closed completely, and patients were immobilized for 3 weeks before starting an early functional rehabilitation protocol. Both of these patients sustained a fall, the first at 13 weeks postoperatively and the second during postoperative week 4. Reruptures occurred at the same site associated with a transverse open wound, and both patients went on to eventual healing with satisfactory results after a second open surgical repair.

The final report of open rerupture was described recently by de Cesar Netto et al. and was the only case of open r-rupture after an initial primary percuteaneous repair [17]. The rerupture occurred 19 weeks postoperatively with no apparent traumatic mechanism. Again, the wound was transverse, perpendicular to the surgical incision, and was eventually rerepaired in an traditional open fashion. This particular case was additionally complicated by infection requiring negative pressure therapy and repeat debridement, but eventually healed.

There are many similarities and a few key differences between these cases of open rerupture. One hypothesis agreed upon by these authors is that the etiology of open rerupture is likely adhesions between the repaired tendon and the subcutaneous tissues causing axial traction on the skin. The two cases described by Garcia-German et al. are unique and highlight two important risk factors for increased adhesion formation which are a bulky repair limiting complete closure of the paratenon and prolonged immobilization. More aggressive early functional rehab protocols have been shown to decrease the overall rate of rerupture [1, 3] and likely help in prevention of this rare complication as well. However, in the other four cases including our own, neither of these risk factors were present. In the two cases described by Hanada et al., there was a clear mechanism of injury; de Cesar Netto described the only case other than our own where the open rerupture occurred with a lack of traumatic mechanism.

Our case highlights a novel technique for dealing with this rare complication, leveraging the preexisting transverse wound to perform a percutaneous repair technique. This technique minimizes surgical dissection and potential for adhesion formation. Additionally, the decision was made to anchor our proximal sutures in the calcaneus rather than an end-to-end repair to further decrease suture bulk around the repair and potential for adhesions. Rerupture after Achilles tendon repair is associated with functional deficits, and worse patient reported outcomes, [14] secondary rerupture rates around 9%, [4, 13], and infection rates from 2.2 to 10.4% [4]. It is unknown whether this small subset of open reruptures does any worse, and due to the small number of cases described in the literature, it is unlikely we will be able to determine with any high degree of certainty the effect of an open rerupture compared to a closed one. In this small review of six cases, five patients went on to have successful outcomes without additional complication, while one patient had their treatment course complicated by infection and additional surgical procedures.
